# Local delivery of corticosteroids in clinical ophthalmology: A review

**DOI:** 10.1111/ceo.13702

**Published:** 2020-01-22

**Authors:** Adrian T. Fung, Tuan Tran, Lyndell L. Lim, Chameen Samarawickrama, Jennifer Arnold, Mark Gillies, Caroline Catt, Logan Mitchell, Andrew Symons, Robert Buttery, Lisa Cottee, Krishna Tumuluri, Paul Beaumont

**Affiliations:** ^1^ Westmead Clinical School Discipline of Clinical Ophthalmology and Eye Health, University of Sydney, Sydney, New South Wales Australia; ^2^ Department of Ophthalmology, Faculty of Medicine and Health Sciences Macquarie University Sydney New South Wales Australia; ^3^ Save Sight Institute Central Clinical School, Discipline of Clinical Ophthalmology and Eye Health, University of Sydney, Sydney, New South Wales Australia; ^4^ Royal Victorian Eye and Ear Hospital Melbourne Victoria Australia; ^5^ Centre for Eye Research Australia Melbourne Victoria Australia; ^6^ University of Melbourne Melbourne Victoria Australia; ^7^ Liverpool Clinical School, Faculty of Medicine University of New South Wales Sydney New South Wales Australia; ^8^ Marsden Eye Specialists Sydney New South Wales Australia; ^9^ Children's Hospital Westmead Westmead New South Wales Australia; ^10^ University of Otago Dunedin New Zealand; ^11^ Royal Melbourne Hospital Melbourne Victoria Australia; ^12^ Melbourne Retina Associates Melbourne Victoria Australia; ^13^ Eye Doctors Mona Vale Sydney New South Wales Australia

**Keywords:** corticosteroid, dexamethasone, Fluocinolone acetonide, prednisolone acetate, triamcinolone acetonide

## Abstract

Locally administered steroids have a long history in ophthalmology for the treatment of inflammatory conditions. Anterior segment conditions tend to be treated with topical steroids whilst posterior segment conditions generally require periocular, intravitreal or systemic administration for penetration. Over recent decades, the clinical applications of periocular steroid delivery have expanded to a wide range of conditions including macular oedema from retino‐vascular conditions. Formulations have been developed with the aim to provide practical, targeted, longer‐term and more efficacious therapy whilst minimizing side effects. Herein, we provide a comprehensive overview of the types of periocular steroid delivery, their clinical applications in ophthalmology and their side effects.

## 1 INTRODUCTION

The first use of corticosteroids in ophthalmology by Gordon and McLean^1^ in the 1950s was a landmark event that revolutionized the management of inflammatory eye disease. The following decades led to further research into the mechanisms and immunological pathways within the eye, as well as the development of various forms of steroid that are locally administered in clinical practice today. Variations in ocular steroid delivery sites, dosages and preparations have all improved efficacy and durability whilst minimizing side effects. Despite development of systemic immunomodulatory (steroid‐sparing) agents and intravitreal monoclonal antibodies, locally administered steroids continue to retain a fundamental role in the management of many ophthalmic diseases. This paper reviews the mechanism of action, preparations, indications and side effects of locally administered steroids.

## 2 STEROID SUBTYPES AND MECHANISM OF ACTION

**Figure 1:**
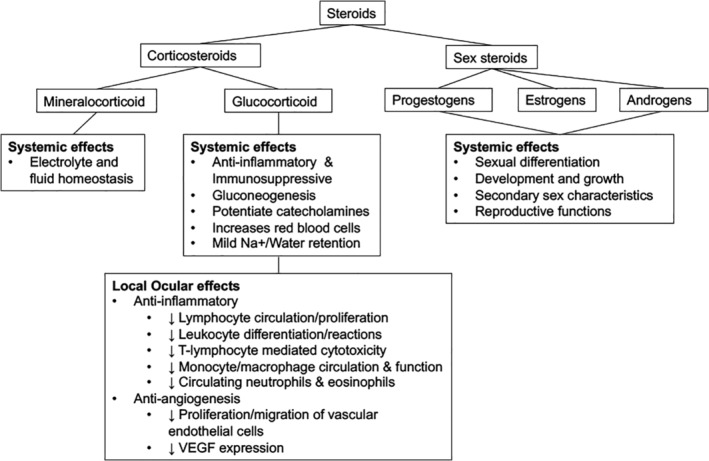
Classification of steroids and actions

Steroids are organic compounds with 17 core carbon atoms bonded in three fused cyclohexane and one fused cyclopentane ring. The main two groups are corticosteroids (glucocorticoids and mineralocorticoids) and sex steroids (progestogens, androgens and estrogens; Figure 1).^2^

Most steroids used in ophthalmology are glucocorticoids, which have anti‐inflammatory and immunosuppressive activity. The synthetic steroid prednisolone has both glucocorticoid and mineralocorticoid receptor activity, whilst the other three main ocular steroids (triamcinolone acetonide [TA], dexamethasone acetonide [DA] and fluocinolone acetonide [FA]) are predominantly active against glucocorticoid receptors (Table 1).^2^

**Table 1:** Potency in receptor activation determined in engineered human HeLa cells^3^

	Glucocorticoid receptor activation potency HeLa cells		Mineralocorticoid receptor activation potency HeLa cells	
	Absolute (nM)	Relative to Cortisol	Absolute (nM)	Relative to cortisol
Short acting				
Cortisol	72	100%	0.04	100%
Intermediate‐acting				
Prednisone/prednisolone	8	900%	0.015	267%
Triamcinolone	1	7200%	>100	<0.04%
Long‐acting				
Dexamethasone	3	2400%	0.3	13%
Fluocinolone	0.4	18 000%	>100	<0.04%

The therapeutic effect of glucocorticoids are mediated via the glucocorticoid receptor in the cytosol which upon activation, undergoes conformational changes and translocate toward the cell nucleus. This activated glucocorticoid receptor signals the transactivation or trans‐repression of gene transcription factors which cause both therapeutic and side effects. There are over 40 distinct isoforms of the glucocorticoid receptor which have varying distribution within the tissues of the eye, each with different downstream signalling effects allowing for diverse cell‐specific actions.^4^

The anti‐inflammatory effect of steroids is caused by inhibiting the transcription of inflammatory and immune genes. These actions block the release of arachidonic acid and its subsequent eicosanoids (prostaglandins, thromboxanes, prostacyclins and leukotrienes).^5^ This affects the blood‐retinal barrier with a reduction in fibroblast proliferation, collagen and scar formation, retinal oedema, fibrin deposition, capillary leakage, intraretinal migration of inflammatory cells and levels of vascular endothelial growth factor (VEGF).

## 3 STEROID PREPARATIONS AND METHODS OF LOCAL ADMINISTRATION TO TREAT OPHTHALMIC DISEASE

**Figure 2:**
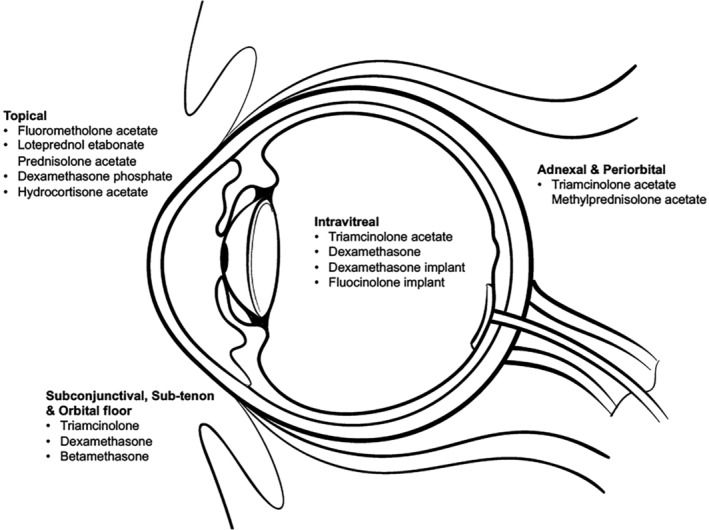
Common locally administered ophthalmic steroids

Glucocorticoids may be locally administered in the following ways: topical, sub‐conjunctival, periocular (sub‐Tenon, orbital floor, peribulbar) and intravitreal (Figure 2). Regional administration allows for high levels of ocular delivery (intravitreal steroid bypasses the blood‐retinal barrier) whilst minimizing systemic side effects. An overview of steroid preparations and their local delivery methods are presented in Table 2.

**Table 2:** Ocular steroid preparations and their delivery sites, generic name (trade name)

1. Topical	Dexamethasone sodium phosphate 0.1% (MAXIDEX, Decadron) Dexamethasone sodium phosphate ointment 0.05% (Dexadron) Prednisolone acetate 1% (Pred Forte, Econopred Plus, AK‐Tate) Prednisolone acetate 0.12% (Pred Mild, Econopred) Prednisolone sodium phosphate 1% (Inflamase Forte, AK‐Pred) Prednisolone sodium phosphate 0.5% (Prednisolone Minims, Metreton) Prednisolone phosphate 0.5%, 0.25% ointment (Hydeltrasol) Fluorometholone alcohol 0.1% or 0.25% suspension (FML Forte, FML, FML liquifilm) Fluorometholone ointment 0.1% (FML SOP) Fluorometholone acetate 0.1% (FLAREX) Hydrocortisone acetate 1% ointment (Siguent Hycor) Medrysone 1% suspension (HMS) Rimexolone 1% (Vexol) Medroxyprogesterone acetate 1% (Provera) Loteprednol etabonate 0.5% (Lotemax, Alrex) Difluprednate 0.05% emulsion (Durezol)
2. Sub‐conjunctival	Hydrocortisone 100 to 1000 mg powder (hydrocortisone sodium succinate) Methylprednisolone sodium succinate 40 mg/mL, 125 mg/mL, 2 g/40 mL solution (Solu‐Medrol) Methylprednisolone acetate 40 mg/mL (Depo‐Medrol) Triamcinolone diacetate 25 to 40 mg/mL suspension (Aristocort) Triamcinolone acetonide 10 to 40 mg/mL suspension (Kenalog, Kenacort‐A 10, Kenacort‐A 40) Triamcinolone acetonide 40 mg/mL (Triescence) Dexamethasone acetate 6 to 16 mg/mL (Decadron‐LA) Betamethasone acetate and sodium phosphate 3 mg/mL suspension (Celestone Soluspan)
3. Periocular (intra‐lesional [eyelids], Juxtascleral, sub‐Tenon, orbital floor, peribulbar)	Hydrocortisone 100 to 1000 mg powder (hydrocortisone sodium succinate) Methylprednisolone sodium succinate 40 mg/mL, 125 mg/mL, 2 g/40 mL solution (Solu‐Medrol) Methylprednisolone acetate 20 to 80 mg/mL (Depo‐Medrol) Triamcinolone diacetate 25 to 40 mg/mL suspension (Aristocort) Triamcinolone acetonide 10 to 40 mg/mL suspension (Kenalog, Kenacort‐A 10, Kenacort‐A 40) Triamcinolone acetonide 40 mg/mL (Triescence) Dexamethasone 0.4 mg implant (Dextenza), inserted into lacrimal puncta Dexamethasone acetate 6 to 16 mg/mL (Decadron‐LA) Dexamethasone sodium phosphate 4, 10, 24 mg/mL solution (Decadron Phosphate) Betamethasone acetate and sodium phosphate 3 mg/mL suspension (Celestone Soluspan)
4. Intravitreal	Triamcinolone acetonide 10 to 40 mg/mL suspension (Kenalog, Kenacort‐A 10, Kenacort‐A 40) Triamcinolone acetonide 40 mg/mL (Triescence) Dexamethasone solution 9% (DEXYCU) Dexamethasone 0.7 mg implant (OZURDEX) Fluocinolone acetonide 0.19 mg implant (Iluvien) Fluocinolone acetonide 0.18 mg implant (Yutiq) Fluocinolone acetonide 0.59 mg implant (Retisert)

### 3.1 Topical

Topical steroids are used to treat inflammation of the conjunctiva, cornea and the anterior segment. In certain circumstances they can also be useful in treating uveitic or postoperative macular oedema. Penetration into the aqueous humour occurs by diffusion across the cornea.^6^

Dexamethasone is approximately 25 to 30 times intrinsically more potent than hydrocortisone.^7^ However, the efficacy of each preparation depends not only on the drug's intrinsic potency, but its penetration and durability. Acetate preparations are more lipophilic than those with phosphate preparations, and hence have greater corneal penetration.^8^ Although prednisolone acetate is six times less potent on a molar basis than dexamethasone or betamethasone, due to the acetate preparation, topical prednisolone acetate 1% provides greater anti‐inflammatory effect than either dexamethasone or betamethasone phosphate 0.1%.^9^ Solutions with preservatives also have greater penetrance than those without, as the preservative disrupts tight junctions between corneal epithelial cells. The frequency of application also increases the anti‐inflammatory effect. A study on corneal inflammation demonstrated greater anti‐inflammatory effects when topical prednisolone acetate is applied every 15 minutes (or five doses at 1 minute intervals each hour) versus hourly.^10^ It is important that suspensions are shaken immediately prior to use, otherwise the administered dosage will vary.

### 3.2 Sub‐conjunctival

Sub‐conjunctival steroids are frequently administered at the conclusion of intraocular surgery. The most common preparation used is dexamethasone, although methylprednisolone may also be given.^11^ Dexamethasone has been shown to achieve good ocular penetration following sub‐conjunctival injection, with higher levels of concentration in the aqueous and vitreous than when it is administered as a peribulbar injection or orally.^12^ Sub‐conjunctival TA has been shown to be efficacious and safe for anterior uveitis and non‐necrotising, non‐infectious anterior scleritis.^13^, ^14^

### 3.3 Periocular

Sub‐Tenon, orbital floor and peribulbar steroids are frequently used to treat ocular inflammatory conditions, particularly when there is associated macular oedema and in whom systemic side effects are less desirable. After 30 days following a single sub‐Tenon injection of 40 mg of TA, corticosteroid levels can be found in all ocular tissues, with highest levels within the choroid and retinal pigment epithelium, whilst systemic levels remain low.^15^ The drug of choice is usually TA, formulations of which include Kenacort and Triesence.

Posterior juxtascleral depot injection of anecortave acetate was previously used to treat choroidal neovascularisation. Anecortave acetate (Retaane) is a synthetic angiostatic steroid that was formulated to be devoid of glucocorticoid receptor‐mediated activity. It was delivered as a posterior juxtascleral depot every 6 months. Although its efficacy in neovascular age‐related macular degeneration (AMD) was shown against placebo,^16^ it did not strongly demonstrate significant benefit against photodynamic therapy with verteporforin.^17^ The role of anecortave acetate was soon superceded by the emergence of intravitreal anti‐VEGF.

### 3.4 Intravitreal

Steroids are most potent against retinal disease when delivered intravitreally. Intravitreal steroids are used for macular oedema, uveitis and to stain the vitreous during intraocular surgery for improved visualization. As the procedure involves globe penetration, it must be done under aseptic conditions. It may be given as an intravitreal injection (Kenacort, Triesence), or as a slow‐release intravitreal implant (OZURDEX, Iluvien, Retisert).

#### 3.4.1 Triamcinolone acetonide

TA is a minimally water‐soluble suspension. After intravitreal injection, triamcinolone crystals slowly dissolve into the vitreous. This creates a diffusional gradient from the vitreous to the macula with minimal systemic exposure. While a portion of the drug targets the macula, another portion either clears through the retina or diffuses to the anterior segment where it can cause cataract or elevation of intraocular pressures (IOPs).

Kenacort was formulated for intra‐articular and intramuscular injection and thus its application in ophthalmology is off‐label. In contrast, Triesence is a preservative‐free preparation of TA. The pharmacokinetics and pharmacodynamics of different TA preparations have been shown to differ in animal studies.^18^, ^19^ Since only dissolved free triamcinolone has a therapeutic effect, durability depends on multiple factors such as pH, particle size (smaller and more uniform for Triesence compared with Kenacort), crystallinity, solubility and dissolution kinetics in the vitreous.^19^ The duration of effect of intravitreal TA (IVTA) lasts between 3^20^ and 6^21^ months in non‐vitrectomised eyes, but is up to six times shorter in vitrectomised eyes.^20^

Kenacort comes in two dosages: Kenacort‐A 10 (10 mg/ml) and Kenacort‐A 40 (40 mg/ml). As its use in ophthalmology is off‐label, no specific dosage is recommended however most studies for diabetic macular oedema (DMO) have injected 4 mg in 0.1 mL. The SCORE studies for macular oedema secondary to retinal vein occlusion showed no significant differences between the 1 mg/0.1 mL and 4 mg/0.1 mL preservative‐free IVTA (Trivaris Allergan, Inc., Irvine, California) arms.^22^, ^23^ The manufacturer of Triesence (Alcon) recommends an initial dosage of 4 mg/0.1 ml for therapeutic purposes, and 1 to 4 mg for visualization during vitrectomy.^24^

#### 3.4.2 Dexamethasone intravitreal implant

OZURDEX is a biodegradable intravitreal implant that contains 0.7 mg dexamethasone in a NOVADUR solid rod‐shaped polymer drug delivery system. It is designed to release drug over 3 to 6 months in a biphasic fashion with higher doses in the initial 6 weeks followed by lower doses for up to 6 months. It is injected using a single‐use intravitreal applicator with a stepped technique. It is used to treat DMO, macular oedema due to branch or central retinal vein occlusion (BRVO, CRVO) and non‐infectious posterior uveitis.

OZURDEX is contraindicated if there is active ocular infection, hypersensitivity to the drug, advanced glaucoma or posterior lens capsule rupture.^25^ In vitrectomised eyes, there may be an advantage in using the dexamethasone intravitreal implant (DII) over other bolus intravitreal therapies which have a reduced half‐life.^20^ Rabbit‐studies have shown no difference in clearance rates of DII in vitrectomised and non‐vitrectomised eyes.^26^ The efficacy of DII has also been shown to be similar in vitrectomised and non‐vitrectomised eyes when used to treat macular oedema secondary to CRVO.^27^

#### 3.4.3 FA implant

Fluocinolone acetonide implants are synthetic corticosteroid with low solubility in aqueous allowing extended drug release.

Iluvien is an injectable intravitreal 0.19 mg implant within a rod‐shaped (3.5 × 0.37 mm) non‐biodegradable reservoir that has a duration of action of 18 to 36 months. The FAMOUS study demonstrated a sustained release by measuring aqueous concentrations, with levels of slightly more than 2 ng/mL for the first 3 months followed by maintained concentrations of 0.5 to 1.0 ng/mL from 6 to 36 months.^28^

Retisert is a non‐biodegradable disc‐shaped intravitreal implant containing 0.59 mg of FA within a silicone elastomer. It is surgically inserted through a pars plana incision and removed by a second surgical procedure. It has a duration of 18 to 30 months with an initial active drug release of 0.6 μg/day to an eventual steady‐state release of 0.3 to 0.4 μg/day for 30 months.^29^

Yutiq is a recently developed intravitreal implant containing 0.18 mg of FA which is designed to deliver sustained release for up to 36 months. Fluocinolone is contained in the core of a polyamide polymeric cylinder (3.5 × 0.37 mm) with a permeable polyvinyl alcohol membrane. It is injected by a pre‐loaded sterile applicator with a 25‐gauge needle. The drug delivers an approximate initial rate of 0.2 μg daily followed by 0.1 μg daily over 36 months.^30^

## 4 USES OF LOCALLY ADMINISTERED STEROIDS

An outline of studies on the local delivery of corticosteroids in clinical ophthalmology is presented in Table 3.

### 4.1 Ocular adnexae‐eyelids, lacrimal gland and orbit

Oculoplastic uses of topical or intra‐lesional steroids is limited to a few conditions.

#### 4.1.1 Thyroid eye disease

Peribulbar and intra‐orbital steroids have been used in management of active thyroid eye disease (TED). Two randomized studies, with small patient numbers, showed reduction in clinical activity score and extraocular muscle size.^31^, ^32^ Both studies required multiple peribulbar injections of TA 20 mg. There were few reported side effects‐only two cases developed raised IOP. Although they can have an adjunctive role in active TED, they are not first‐line therapy.

Peribulbar and sub‐conjunctival steroids have also been used for upper eyelid retraction in TED.^33^, ^34^, ^35^, ^36^ Studies show that repeat injections of 20 mg of TA are required (one to four injections at monthly intervals) and the response rate is better in patients with recent onset of upper lid retraction or active disease. Xu et al^37^ noted an improvement in 83.3% of patients with symptom duration less than 6 months, compared to 36.4% who responded if symptoms were greater than 6 months. Joos et al^36^ showed that a superior orbital peri‐levator injection technique improved lid retraction and demonstrated reduction in size of the levator/superior rectus complex on MRI imaging after repeat injections.

#### 4.1.2 Histiocytic orbital lesions

Histiocytic lesions are divided into Langerhans cell histiocytosis (LCH) and non‐LCH lesions, of which Juvenile Xanthogranuloma (JXG) is the most common. Langerhans cell histiocytosis can be monofocal (often seen in the frontal bone of the orbit), or systemic. It often presents in children, but can occur in all age groups. It results in bone destruction and secondary soft tissue expansion.^38^ The treatment of choice for orbital LCH is incisional biopsy with curettage of the lesion and intra‐lesional steroids (triamcinolone 40 mg/mL or methylprednisone).^39^, ^40^ JXG often presents with cutaneous involvement. Eyelid and orbital lesions are rare and can be managed with a combination of intra‐lesional steroids with or without surgical debulking.^41^

#### 4.1.3 Periorbital capillary haemangiomas

The treatment of choice for periorbital infantile haemangiomas has traditionally been intra‐lesional and systemic steroids. In 2008 Léaute‐Labrèze et al^42^ published a series of 11 cases of infantile haemangioma cases managed with propranolol which has revolutionized treatment. Two systematic reviews show both intra‐lesional steroids and propranolol are effective, though less side effects occur with propranolol.^37^, ^43^ Propranolol is now the mainstay of treatment for periorbital infantile haemangioma and intra‐lesional steroids are used as an adjunct in resistant cases.

#### 4.1.4 Nasolacrimal disease

Topical steroid drops and steroid nasal spray have been used in management of nasolacrimal duct obstruction (NLDO), with little evidence for their use. In functional NLDO with associated symptoms of rhinitis, topical steroid nasal spray (eg, mometasone and budesonide) may improve epiphora.^44^ The anecdotal improvement of epiphora and mucus discharge in complete NLDO with topical steroid drops is likely secondary to the anti‐inflammatory effect and reduced mucus in the lacrimal sac. Mansur et al^45^ recently assessed the lacrimal complications associated with systemic chemotherapy agents and suggested minor canalicular blockages may be effectively treated with probing and topical steroid drops. If there is a more significant blockage or likely long‐term chemotherapy, then lacrimal surgery is advised.^45^

#### 4.1.5 Chalazia

Traditionally non‐resolving or large chalazia of the eyelids are treated by surgical incision. However, studies show equally effective results with 0.2 to 0.4 mL of 10 mg/mL intra‐lesional triamcinolone.^46^, ^47^ A meta‐analysis of randomized studies showed incision and curettage was more effective than steroid injection as a single procedure, but with repeat procedures similar outcomes were shown.^48^ Intra‐lesional steroid for chalazia is an acceptable treatment for primary and recurrent chalazia.

#### 4.1.6 Periocular scarring

Hypertrophic scarring following surgery or trauma has traditionally been managed with a mixture of topical or intra‐lesional steroids or surgery. Recently, combination therapy of intra‐lesional triamcinolone and 5‐FU has shown great promise in improving periocular scarring post‐surgery.^49^

### 4.2 Anterior segment

Steroids are used frequently in anterior segment diseases,^4^ however there is a considerable lack of randomized control trials (RCTs) to guide treatment.

#### 4.2.1 Corneal transplants

Corticosteroids are the principle medication in the management of corneal transplantation. They are readily absorbed through the cornea and achieve a high concentration in the anterior chamber through topical application. Prednisolone and dexamethasone are the most commonly used forms.^50^ Multiple treatment regimes exist, but as a guide prednisolone 1% or dexamethasone 0.1% drops are used every 2 hours initially, tapered over a period of 6 to 12 months, and a mild steroid used daily for maintenance treatment in endothelial keratoplasties.

Some have advocated for the use of steroids prior to high‐risk transplantation^50^, ^51^, ^52^, ^53^ but this has been variably adopted. A survey of the Bowman's Club (The UK Society of corneal surgeons) found that topical dexamethasone was used in 33%, oral prednisolone by 22% and single dose IV methylprednisolone (IVMP) by 14%.^54^

During corneal allograft rejection, topical, sub‐conjunctival, periocular and/or systemic corticosteroid use is the treatment of choice, with the majority of corneal specialists favouring topical formulations (with a preference for prednisolone acetate 1%).^55^, ^56^ In severe cases of allograft rejection oral or IVMP is often added, and one prospective study suggested that a single dose of 500 mg of IVMP is more effective and better tolerated than daily oral prednisolone.^57^ Unfortunately there are no RCTs to add weight to this study.

#### 4.2.2 Bacterial keratitis

The greatest evidence for the use of steroids in bacterial keratitis come from the Steroids for Corneal Ulcers Trials (SCUT).^58^, ^59^, ^60^ A cochrane review of steroid use in bacterial keratitis^61^ found four RCTs that met inclusion criteria, but only the SCUT trial was of sufficient power to determine the effect of steroids in bacterial keratitis.

This SCUT trial examined the outcomes of 500 cases of culture‐positive bacterial keratitis where fungal, acanthamoeba, HSV, impending perforation and previous corneal transplant patients were all excluded. All cases received moxifloxacin q1h for 48 hours prior to randomization; at randomization, half the patients received prednisolone 1% for a total of 3 weeks only (QID for 1 week, BD for 1 week and daily for 1 week) compared to placebo. In both 3 and 12 months reports, there were no difference between groups in any parameters measured (best‐subjective corrected visual acuity [BSCVA], scar size, rate of re‐epithelialization, rate of perforation). This report added to the weight of evidence that steroids do not cause corneal perforation in bacterial keratitis. The IOP was lower in the steroid group at 3 months as inflammation was better controlled (*p* = .04).

However, subsequent subgroup analysis demonstrated a benefit for the use of steroids. In the 3‐month report, those with baseline BSCVA of CF or worse and those with an infiltrate covering the central 4 mm of the cornea performed better with early introduction of steroids compared to placebo (a two‐line difference in vision, *p* < .05) indicating that there is a benefit of steroids in severe, central infections in the early stage of recovery. At 12 months, when Nocardia infections were removed from the cohort, those who had steroids after 48 hours of antibiotic treatment had a one‐line improvement in BSCVA compared to those who did not have steroids.

#### 4.2.3 Herpes simplex keratitis

Steroid use in herpes simples keratitis (HSK) is mainly for stromal and endothelial keratitis. Much of the evidence for the use of steroids in HSK comes from the double blind, placebo‐controlled RCT known as the Herpetic Eye Disease Study (HEDS).^62^ The HEDS demonstrated a clear benefit of the use of topical prednisolone in the treatment of stromal keratitis.^62^ Those on trifluridine plus prednisolone had a treatment failure rate of 26% compared to 73% on trifluridine plus placebo (*p* < .001). The study also demonstrated that a 10‐week tapering course of steroids was too brief as 50% developed a recurrence within 6 weeks. Thus, for non‐necrotising stromal keratitis without an epithelial defect, antiviral treatment in conjunction with topical steroids for at least 10 weeks is recommended.

Endothelial disease typically presents independently of other forms of HSV keratitis and only few studies are available to guide treatment.^63^, ^64^, ^65^ These compare topical betamethasone with topical acyclovir against topical acyclovir alone (all five times a day) and found that the addition of steroid resulted in a faster response and fewer treatment failures than antiviral alone. Thus, the recommendation for HSV endothelial disease is the combination of antiviral treatments with topical steroids, tapered according to patient signs and symptoms.

#### 4.2.4 Allergic eye diseases

Allergic eye diseases cover a spectrum from seasonal allergic disease through to vernal keratoconjunctivitis (VKC) and atopic keratoconjunctivitis. Corticosteroids play an important role in controlling acute exacerbations; however, they should not be used as long‐term maintenance due to their side effects.^66^ In children with severe VKC, intraocular pressure rises have been reported in up to 28.3% of patients, with 5.5% progressing to glaucoma.^67^ Various regimes of topical steroids can be employed depending on severity of disease with early introduction of a steroid‐sparing agent when the patient is expected to require long‐term disease control.

Supratarsal injection of steroid is effective in refractory, severe and challenging cases of allergic eye diseases.^68^ Two prospective, randomized, double‐masked, case‐control trials showed no difference between dexamethasone sodium (2 mg) phosphate, TA (10‐20 mg) and hydrocortisone sodium succinate (50 mg) in improving severe refractory VKC with resolution of many symptoms by 3 weeks.^69^, ^70^ However, symptoms recurred about 12 weeks post‐treatment without anti‐allergy medication.^69^

#### 4.2.5 Corneal neovascularization

Topical steroids are the mainstay of treatment for the suppression of early proliferating corneal vessels.^71^, ^72^, ^73^, ^74^, ^75^ They act primarily due to suppression of inflammation associated with new vessels and are not necessarily angio‐regressive.^76^ As such, steroids are most effective when applied before, or immediately after corneal injury.^71^

#### 4.2.6 Keratoconjunctivitis sicca

Topical steroids have a role for treating keratoconjunctivitis sicca (KCS), as outlined in the Tear Film and Ocular Surface Society Dry Eye Workshop II (TFOS DEWS II) report.^77^ This report summarizes the currently available evidence on managing dry eye disease, including results from several RCTs,^78^, ^79^, ^80^, ^81^, ^82^, ^83^, ^84^, ^85^, ^86^, ^87^ and concluded that short courses of corticosteroid are effective in improving symptoms of KCS. However, this is not an effective long‐term strategy due to potential side effects. Typically, low strength steroids such as FML were used QID.

#### 4.2.7 Graft vs host disease

Ocular involvement of Graft Versus Host Disease (GVHD) may cause an acute or chronic immunologically mediated inflammatory disease of the ocular surface. Whilst systemic corticosteroids are the mainstay of controlling the acute exacerbations of chronic GVHD, adjunctive topical steroids is often used to allow tapering and cessation of systemic immunosuppression. Small series have retrospectively demonstrated efficacy of topical steroid treatment in controlling acute conjunctival inflammation and reducing scarring, however signs of KCS remained.^88^

Long‐term topical steroids are not recommended after the acute inflammatory phase, when other anti‐inflammatory agents, such as cyclosporin A and tacrolimus may be employed. This is supported by a recent RCT of 42 patients that assessed dry eye disease in chronic GVHD. Topical loteprednol etabonate 0.5% was found to have a minimal effect in ocular surface disease index (OSDI) and corneal fluorescein staining compared to topical lubricants.^89^

#### 4.2.8 Cicatrising conjunctival disorders

##### Chemical and thermal injury

The goal of therapy following chemical and thermal anterior segment injuries is to restore the ocular surface and maintain long‐term corneal clarity by preventing cicatrisation and limbal stem‐cell deficiency. Along with other important aspects of treatment, topical steroids may be used to limit the profound associated inflammation and promote healing. However, there has been controversy regarding the use and timing of topical corticosteroids.

Corticosteroids may have beneficial effects on inflammatory cell suppression and collagenase inhibition, however they may also suppress keratocyte migration and collagen production and thus cause corneal thinning.^90^ Generally, their anti‐inflammatory effect is maximal in the first week after which they should be tapered due to the risk of corneal thinning. Their use alone has been cautioned as it has been shown to cause further corneoscleral melt.^91^ Monitoring for infection or prophylactically adding topical antibiotics prior to epithelialisation is also recommended. One study found a risk of corneoscleral melting if topical steroids were used after 10 days of the chemical injury. The timing coincides where suppression of keratocyte collagen production by corticosteroids may outweigh the advantages of inflammatory cells suppression and collagenase inhibition to promote corneal ulceration.^90^ Davis et al and Brodovsky et al, found in their retrospective series that the prolonged use of topical prednisolone 0.5% used concurrently with topical ascorbate 10% was not associated with corneoscleral melt.^92^, ^93^

##### Ocular cicatricial pemphigoid/mucous membrane pemphigoid

Mucous membrane pemphigoid is a systemic disease primarily affecting mucous membranes. When localized to the conjunctiva, the condition is known as ocular cicatricial pemphigoid. It manifests as a chronically progressive conjunctival inflammation causing bilateral blindness. Systemic immunosuppression is required to halt the progressive inflammation and achieve adequate long‐term remission.^94^ Topical and sub‐conjunctival corticosteroids are used adjunctively with systemic therapy. They may offer short‐term relief of symptoms but are not effective in halting progression of the systemic autoimmune disease.^95^ Due to the infrequency and nature of the condition, there have been no studies assessing their role. Other topical agents shown to give variable results include: calcineurin inhibitors, cyclosporine A, tacrolimus and mitomycin C.^94^

#### 4.2.9 Anterior uveitis

Topical corticosteroids are the mainstay of treating uncomplicated anterior uveitis as it has fewer local and systemic side effects compared to periocular or systemic administration. The interval of drop instillation is tailored to each patient; however, it is generally initiated on frequent intervals then slowly tapered according to the clinical response to prevent rebound inflammation. Where anterior uveitis has not adequately responded to topical corticosteroids, periocular steroids such as sub‐conjunctival dexamethasone may provide greater therapeutic effect with a short duration of action of 1 to 2 days.^96^ Similarly, sub‐conjunctival triamcinolone or betamethasone has also been shown to be safe and effective in severe cases of anterior uveitis.^14^

#### 4.2.10 Non‐necrotising, non‐infectious anterior scleritis

Sub‐conjunctival TA may be given for the treatment of non‐necrotising, non‐infectious anterior scleritis.^13^, ^97^

### 4.3 Glaucoma surgery

The use of corticosteroids in modulating conjunctival wound healing is essential in glaucoma surgery. Topical corticosteroids are routinely used postoperatively and their frequency is often titrated according to the desired effect on wound healing. Sub‐conjunctival corticosteroids are also often injected at the end of surgery, though not usually at the surgical filtration site.

#### 4.3.1 Glaucoma filtration surgery

Glaucoma filtration surgery is aimed at creating a filtering bleb which allows aqueous drainage and thus lowers the IOP. The long‐term success of surgery is dependent on modulating wound healing at the site of filtration, namely the scleral flap and overlying conjunctiva and Tenon's capsule.

Topical postoperative corticosteroids after trabeculectomy have been widely used since the apparent effect on filtering blebs was first described in 1965.^98^ The beneficial effects of steroids after trabeculectomy were first demonstrated in a prospective study in 1985, before the revolutionary widespread use of adjunctive local antimetabolites.^99^ Forty‐six eyes of 35 patients with a diagnosis of primary open‐angle glaucoma or primary angle‐closure glaucoma underwent trabeculectomy were randomized into three groups. Group 1 received no additional steroids, group 2 received topical 1% prednisolone acetate initially every 3 hours then tapered over 20 days, group 3 received the same treatment as in group 2 with additional of oral prednisone (80 mg daily) with a progressive taper over 16 days. The results were followed after 1.5 years, and long‐term data were later published at 5 and 10 years on 58 and 46 eyes, respectively. At 10 years, patients in group 1 (who did not have steroids) had a significantly higher rate (66.7%) of additional glaucoma procedures compared to those in group 2 (11.1%) and group 3 (38.5%). Furthermore, patients in group 1 had higher IOPs, were treated with more glaucoma drops and had lower rate of stabilized glaucoma (based on optic disc photography and visual fields).^100^

Perioperative injection of sub‐conjunctival corticosteroids at the filtering site have been demonstrated to give favourable bleb formation and IOP control in a small pilot series,^101^ In contrast, the only prospective randomized study comparing postoperative topical steroids to postoperative topical steroids in addition to steroid depot injection of TA found no significant differences in outcomes.^102^ Nevertheless, since the introduction of locally administered antimetabolites such as 5‐fluorouracil and mitomycin C were found to be more potent in impairing wound healing and thus increase long‐term success rates of trabeculectomy, the potential role of perioperative sub‐conjunctival steroid administration had become less significant.^103^

Currently, locally administered antimetabolite therapy is routinely used in conjunction with topical postoperative corticosteroids to modulate conjunctival wound healing in glaucoma filtration surgery. Corticosteroids predominantly modulate wound healing by reducing the release of acute inflammatory mediators and fibroblast recruitment. They also have a lesser effect in the proliferative phase of wound healing by limiting fibroblast activity. In contrast, antimetabolites modulate wound healing by inhibiting proliferation of fibroblasts and their profibrotic mechanisms.^104^, ^105^ Modulating both the inflammatory and proliferative phases of the wound healing response with these agents increases the likelihood of long‐term filtration and lower postoperative IOPs.^106^

#### 4.3.2 Aqueous shunt surgery

Modulation of wound healing is important in the process of bleb encapsulation in aqueous shunt surgery. The use of corticosteroids to control postoperative inflammation is thought to influence the hypertensive phase after glaucoma drainage implantation. The hypertensive phase is characterized by a rise in IOP due to bleb encapsulation or capsular fibrosis that occurs at approximately 1 to 3 months postoperatively.^107^ It is particularly observed after implantation of non‐valved glaucoma drainage devices such as the Ahmed glaucoma device where it may occur in 56% to 82%.^108^ It has been reported that in 72% of these cases, the elevated IOP does not resolve indicating early surgical failure.^109^

Turalba and Pasquale^110^ retrospectively compared patients who received intraoperative sub‐Tenon during Ahmed device implantation with those who did not. The hypertensive phase was found to be 26% in those who received triamcinolone compared to those without (52%). There was no difference in final IOP outcomes and the authors warned of a higher rate of early complications including tube erosion and endophthalmitis. Yadnazi et al^111^ demonstrated in a prospective randomized trial of 90 eyes that adjunctive sub‐Tenon TA during Ahmed valve implantation had significantly lower IOPs at 1 month and lower peak IOPs, however had no difference in the rates of success or incidence of a hypertensive phase.

### 4.4 Posterior segment

Ocular steroids are used to treat macular oedema of varied aetiology (diabetic, retinal vein occlusion, postoperative and inflammatory), intraocular inflammation including uveitis and scleritis and to assist visualization of the vitreous during vitrectomy.

#### 4.4.1 Diabetic macular oedema

Diabetic macular oedema is the most common cause of vision loss in people with diabetes.^112^ The pathogenesis of DMO is multifactorial, with not only VEGF but other pro‐inflammatory factors involved in breaking down the blood‐retinal barrier and increasing vascular permeability.^113^ Although intravitreal anti‐VEGF therapy remains first‐line treatment for centre‐involving DMO in phakic patients, ocular steroids may be considered for pseudophakic patients, those with planned cataract surgery, or in patients with suboptimal response or contraindication to intravitreal anti‐VEGF therapy.^114^ A suboptimal response to intravitreal anti‐VEGF therapy is not uncommon. In the RISE/RIDE registration trials, BCVA was worse than 6/12 in 42.8% of patients and central foveal thickness greater than 250 μm in 24.8% of patients despite 2 years of monthly intravitreal ranibizumab (0.3 mg) injections.^25^

##### Intravitreal TA for diabetic macular oedema

Intravitreal TA (Kenacort‐A 40, Bristol‐Myers Squibb Pharmaceuticals, Noble Park, Australia) was first shown to be superior to sham treatment for BCVA in patients with centre‐involving DMO in a prospective, double‐masked, placebo‐controlled randomized controlled trial.^115^ Compared with those receiving placebo, eyes receiving TA had a 5.7 Logarithm of Minimum Angle of Resolution (LogMAR) letter better improvement at 2 years.

The Diabetic Retinopathy Clinical Research Network (DRCR.Net) Protocol I study was a multi‐centre RCT comparing sham injection + prompt laser, 0.5 mg ranibizumab + prompt laser, 0.5 mg ranibizumab + deferred laser and 4 mg TA + prompt laser for centre‐involving DMO.^116^ The main outcome measure was improvement in BCVA at 1 year, and 854 study eyes of 691 patients were enrolled. The ranibizumab arms showed greater improvement in BCVA compared with laser alone. Although overall the TA + prompt laser arm did not do as well as the ranibizumab arms, TA was shown to be as effective as ranibizumab when only pseudophakic patients were analysed. However, at 5‐year follow‐up the TA arm was inferior to ranibizumab arms, even when only analysing pseudophakic patients and allowing for the addition of “very deferred ranibizumab” after 74 weeks from baseline.^117^

##### DII for diabetic macular oedema

The MEAD study included two 3‐year, multi‐centre, masked, randomized controlled phase III clinical trials that compared a minimum of 6‐monthly dosing with DII 0.7 mg, 0.35 mg and sham procedure. Patients had centre‐involving DMO, visual acuities between 6/15 and 6/60 and central retinal thickness ≥300 μm on optical coherence tomography scans.^118^ The main outcome measures were proportion of patients achieving ≥15 LogMAR letters of improvement in BCVA and safety profile. At baseline there were 1048 patients enrolled, and 57.9% completed the 3‐year study. Both DII doses had a significantly greater proportion of patients achieving ≥15‐letters of improvement in BCVA (22.2% for 0.7 mg, 18.4% for 0.35 mg and 12.0% for sham).

The BEVORDEX study was a prospective, multi‐centre, randomized single‐masked clinical trial comparing 4‐weekly bevacizumab and DII 0.7 mg (OZURDEX) that could be given more frequently (4‐monthly) in 88 eyes of 61 patients with centre‐involving DMO.^119^ The main outcome measure was the proportion of patients achieving an improvement in vision of 10 LogMAR letters. Each arm had similar proportion of patients reaching the main outcome measure at 12 and 24^120^ months (40% with bevacizumab and 41% with DII at 12 months). The group receiving DII had fewer mean injections (2.7) compared to the bevacizumab arm (8.6) over the first 12 months with a greater reduction in central macular thickness at 12 but not 24 months. However, more patients in the DII arm lost vision, mainly because of cataract progression.

##### FA intravitreal implant for diabetic macular oedema

Iluvien is a sustained delivery FA injectable implant that has been shown to treat patients with DMO. The FAME A and B studies were identically designed parallel‐group, phase 3 double‐masked, randomized controlled phase III clinical trials that compared two doses (0.2 and 0.5 μg/day) of FA over a 3‐year period. The primary end point was a gain of ≥15 letters at 24 months with follow‐up to 36 months.

A pre‐planned subgroup analysis examined visual outcomes as a function of duration of DMO at randomization revealed that the treatment effect resided primarily in patients with chronic DMO (duration ≥3 years). At month 36, a significantly higher proportion of FA treated patients from both studies showed an improvement of ≥15 letters from baseline compared to the sham group (FAME A: 31.8% for 0.2 μg/day, 13.5% for sham; FAME B: 36.4% for 0.2 μg/day, 13.2% for sham). In patients with non‐chronic DMO (duration <3 years), the proportion of patients gaining ≥15 letters were similar between the FA and sham groups in both studies.^121^ In Europe and in the United Kingdom, it has been approved for the treatment of persistent DMO that has not sufficiently responded to available therapies. In the USA, it is approved for the treatment of DMO in patients who have been previously treated with corticosteroids without a clinically significant rise in IOP.

#### 4.4.2 Macular oedema secondary to retinal vein occlusion

Macular oedema is the most common cause of visual loss in RVO.^22^, ^122^, ^123^ Like DMO, anti‐VEGF therapy remains first‐line treatment in phakic patients. This is based on multiple RCTs demonstrating visual benefit of anti‐VEGF for macular oedema secondary to CRVO (ranibizumab: CRUISE^124^, ^125^; aflibercept: COPERNICUS,^126^ GALILEO,^127^ SCORE2^128^; bevacizumab: SCORE2,^128^ Epstein et al^129^) and BRVO (ranibizumab: BRAVO^130^, ^131^; aflibercept: VIBRANT^132^, ^133^; bevacizumab^134^).

##### Intravitreal TA for retinal vein occlusion

Ocular steroids may be considered in pseudophakic eyes in which anti‐VEGF is contraindicated or failing to provide an adequate result.^135^ Ocular steroids not only inhibit VEGF, but their anti‐inflammatory and neuroprotective effects may also benefit eyes with RVO.^22^, ^123^ The Standard Care versus Corticosteroid for Retinal Vein Occlusion (SCORE) studies were multi‐centre randomized clinical trials evaluating the benefit of IVTA for the treatment of macular oedema secondary to retinal vein occlusion.^22^, ^23^ In the SCORE‐CRVO Study (Report 5), 271 patients were randomized to observation (the standard of care at that time), 1 or 4 mg preservative‐free IVTA (Trivaris).^22^ The main outcome measure was the proportion of patients with ≥15 letter improvement from baseline to month 12. This was achieved in significantly more patients on 1 or 4 mg triamcinolone (27 and 26%, respectively) than those who were observed (7%). For the SCORE‐BRVO Study (Report 6) the observation arm was replaced with grid photocoagulation because grid laser was the standard of care at that time for treating macular oedema secondary to BRVO according to the Branch Vein Occlusion Study.^136^, ^137^ Unlike SCORE‐CRVO, there was no significant difference between the arms in the proportion of patients achieving a ≥15 letter improvement from baseline to month 12.

##### Intravitreal dexamethasone implant for retinal vein occlusion

The Global Evaluation of Implantable Dexamethasone in Retinal Vein Occlusion with Macular Edema (GENEVA) study included two identical multi‐centre, masked, 6‐month, sham‐controlled RCTs assessing the efficacy of DII implant for vision loss due to macular oedema from both CRVO and BRVO.^123^ A total of 1267 patients were randomized to receive a single treatment of DII 0.7, 0.35 mg, or sham procedure. Both DII groups performed significantly better than the sham arm in the time to reach a ≥15 letter improvement in BCVA, proportion of patients achieving a ≥15 letter improvement in BCVA, mean BCVA and proportion of patients losing ≥15 letters.

##### Intravitreal fluocinolone implant for retinal vein occlusion

There is a lack of studies on intravitreal FA implants for the treatment of CMO from retinal vein occlusions. The Fluocinolone Acetonide Intravitreal Inserts for Vein Occlusion in Retina (FAVOUR) study started recruiting patients however the study was terminated early. Currently, Iluvien has not been approved for macular oedema from retinal vein occlusions in the USA, UK or Europe and any use for this indication is off‐label.

Retisert has been used for CMO in retinal vein occlusions within a small pilot series by Ramachandran et al^138^ which demonstrated 69% of eyes showing visual acuity improvement, 15% were stable and 15% lost two lines from baseline at 12 months. Cataract formation occurred in almost all patients and 39% eyes required glaucoma filtration surgery by 12 months. A follow‐up study recruited 10 further patients and indicated sustained benefit up to 30 months.^139^

#### 4.4.3 Posterior non‐infectious uveitis

For patients with posterior segment inflammation and macular oedema, topical steroid therapy is often inadequate. These patients have the option of periocular (sub‐Tenon, orbital floor, peribulbar), intravitreal (IVTA, OZURDEX) and systemic steroids. In an attempt to minimize the systemic side effects (such as Cushingoid state, osteoporosis and elevated blood glucose), local steroid is often considered, especially for unilateral inflammation. In the retrospective cohort of the Systemic Immunosuppressive Therapy for Eye Diseases (SITE) study, over half of 1192 eyes in 914 patients with uveitis demonstrated improved visual acuity at some point within 6 months of receiving periocular steroid.^140^

Intravitreal steroids are particularly useful in two groups of patients: those with severe vitritis or cystoid macular oedema (CMO) that is unlikely to respond rapidly to periocular corticosteroids, and those with inflammation that are refractory to other treatment. In patients with persistent disease, these options may also be combined effectively with systemic steroids and steroid‐sparing agents (eg, methotrexate, mycophenolate and cyclosporin) for acute unilateral relapses or persistent disease activity to reduce the dosages and side effects of the systemic treatment.

It should also be noted that although ongoing repeated depot steroid injections could be considered as a treatment option for chronic persistent intermediate, posterior or panuveitis, such a management approach must be considered with caution given the recent 7‐year follow‐up findings from the NIH sponsored MUST (Multi‐centre Uveitis Steroid Treatment) trial. This study was also a prospective RCT that compared the FA containing Retisert implant with standard systemic immunosuppression in 479 eyes. Although the implant group initially had a faster gain in BCVA, the systemic treatment group had a more gradual gain in BCVA such that there was no significant difference at 2 and 5 years.^141^ However, at 7 years, the systemic group overtook the implant group in terms of BCVA outcomes,^142^ with the implication being that the uveitis relapses occurring once the depot steroid “wears off” are more severe and more likely to result in more (irreversible) damage than lower grade relapses seen with systemic treatment when oral prednisolone/immunosuppression is being gradually weaned.

A prospective 3‐year randomized, sham‐controlled study is comparing Yutiq with placebo. Yutiq is designed to release FA for up to 36 months and the 12‐month data has shown it was effective in lowering the rate of recurrence of posterior uveitis. At 36‐months, the effect of reducing recurrence rate was still significantly lower with Yutiq (56.3%) compared to sham‐treated eyes (92.9%).^30^

#### 4.4.4 Uveitic macular oedema

Cystoid macular oedema is a common cause of vision loss in uveitis.^24^ Intravitreal TA has been shown to effectively reduce uveitic CMO.^143^ Visual acuity improvements are more significant if the CMO is present for 12 months or less and for patients aged 60 years or younger. It is useful in improving visual acuity in patients with CMO, even when their non‐infectious uveitis has been quiescent.^144^ As a single IVTA injection lasts approximately 3 to 6 months, repeated injections may be required.^145^

Alternatives to IVTA for treating uveitis include DII or FA implants. The HURON trial demonstrated efficacy of a single OZURDEX injection in non‐infectious intermediate, posterior or panuveitis in comparison to placebo, with a reduction of inflammation and CMO in 47%, and ≥15 letter gain in up to 43%.^146^

Most recently, the National Institute of Health (NIH) funded POINT trial compared all three of the above depot steroid options (periocular TA, IVTA and OZURDEX) for the treatment of CMO secondary to uveitis in a prospective, multi‐centre RCT.^147^ In this trial, 235 eyes were randomized 1:1:1 to either periocular TA (40mg/1ml), unpreserved IVTA (4mg/0.1ml) or OZURDEX (0.7mg dexamethasone). The primary outcome was central subfield macular thickness (CMT) at 8 weeks, with secondary outcomes including visual acuity and rate of adverse events over 24 weeks of follow‐up. Overall, the CMT in all three groups improved, however IVTA and OZURDEX were found to be superior to periocular TA, with rates of improvement of 39% and 46%, respectively, vs 23% for periocular TA (*p* < .0001). However, no statistically significant difference was demonstrated between IVTA and OZURDEX. Similarly, BCVA also improved in all three groups, with greater gains (four to seven letters to more greater gains) seen with intravitreal treatments, with again no clinical or statistically significant differences seen between IVTA or OZURDEX. Interestingly, despite the findings in other studies, the duration of effect of OZURDEX on CMT was found to decrease after 8 weeks (rather than 12‐16 weeks) in this cohort of patients. This may indicate that patients with uveitic CMO may require intravitreal injections more frequently than for other indications. It should be noted that the POINT study design did allow the IVTA arm to have re‐treatments at 8 weeks, but only at 12 weeks for the OZURDEX arm.

#### 4.4.5 Bacterial endophthalmitis

Intravitreal steroids have been described for the management of acute bacterial endophthalmitis in conjunction with intravitreal antibiotics, although their use remains controversial.^148^, ^149^ They may tamper the inflammatory response that causes damage to the retina, but, conversely, they may interfere with infection control, lower the concentration of intravitreal antibiotics and the additional volume may elevate the IOP.^149^ If they are used, intravitreal dexamethasone is preferred due to its rapid elimination from the eye.^149^ A dosage of 0.4 mg/0.1 mL is usually prescribed, as higher doses have been shown to cause Müller cell damage in animal studies.^150^ Evidence for intravitreal dexamethasone in acute endophthalmitis is limited to retrospective case series which gave mixed results, and four prospective RCTs^151^, ^152^, ^153^, ^154^ which failed to show statistically significant improvements in final visual outcomes.^149^ Although traditional teaching is to avoid intraocular steroids for fungal endophthalmitis, this fear may be exaggerated in patients treated with vitrectomy and intravitreal anti‐fungal therapy.^155^

#### 4.4.6 Postoperative macular oedema

Macular oedema is a well‐known complication of cataract surgery. Topical non‐steroidal and steroidal therapy are usually first‐line treatment.^156^ Topical and oral carbonic anhydrase inhibitors and intravitreal anti‐VEGF agents have also been described, but strong evidence for these are lacking. In recalcitrant cases, local steroid injections may be considered. IVTA has been shown to reduce retinal thickness and improve vision in cases of persistent pseudophakic macular oedema^157^, ^158^, ^159^, ^160^ with an effect that may be sustained for more than 6 months.^158^

#### 4.4.7 Other indications for intravitreal TA and dexamethasone implant

Intravitreal steroids have been used with variable results for a variety of other causes of macular oedema including: neovascular AMD,^161^ retinal angiomatous proliferation,^162^ macular telangiectasia,^163^, ^164^ Coat's disease,^165^ vasoproliferative tumour,^166^ radiation retinopathy,^167^, ^168^ retinitis pigmentosa,^169^, ^170^ proliferative vitreoretinopathy,^171^, ^172^, ^173^ following scleral buckling^174^ or vitrectomy surgery^175^ and from idiopathic CMO.^160^^,^ 176

Intravitreal TA can be used intraoperatively to visualize the vitreous. This is particularly useful for iatrogenic induction of a posterior vitreous detachment, peeling internal limiting membrane and when vitreous needs to be highlighted for clearance in complicated cataract surgery.^177^

## 5 COMPLICATIONS OF OCULAR AND PERIOCULAR STEROID DELIVERY

Complications arising from use of ocular steroids may be related to the procedure itself, or the pharmacological effects of the steroids.

### 5.1 Procedure related complications

#### 5.1.1 Periocular injections

Periocular injections can be performed using different techniques: into the sub‐conjunctival space, into the sub‐Tenon space, into the orbital floor alongside the globe, (usually inferiorly, via a transcutaneous or transconjunctival injection), or into the peribulbar or retrobulbar space. Complications of these injections include: orbital swelling, chemosis, proptosis, sub‐conjunctival haemorrhage, retrobulbar haemorrhage, globe ischaemia, posterior ischaemic optic neuropathy, optic atrophy, globe perforation, orbital cellulitis, fat atrophy, fat herniation, damage to the rectus muscles resulting in diplopia, ptosis, dural puncture and an oculocardiac reflex.^178^, ^179^, ^180^, ^181^

The likelihood of complications differs depending on the site of the injection. Posterior injection reduces the chances of unsightly sub‐conjunctival plaques resulting from anterior seepage of depot, conjunctival or corneoscleral melting, depigmentation and granuloma related to the methylcellulose vehicle of the depot injection. Injection into the orbital floor is easily performed with a 25 mm 25‐gauge needle. It is well tolerated and carries only a very small risk of globe perforation if the needle is directed away from the globe at all times. It is frequently difficult to access the sub‐Tenon's space of patients who have had previous surgery (notably scleral buckling), these eyes may be more suited to an orbital floor injection. Peribulbar and retrobulbar injection are more likely to lead to globe perforation or inadvertent intravascular injection with vascular occlusion from embolization. Additional caution is required in myopic patients as they have a thinner sclera and larger globes which are at an increased risk of perforation.

#### 5.1.2 Intravitreal injections

Intravitreal injections may be associated with endophthalmitis, ocular inflammation, vitreous haemorrhage, retinal tears, rhegmatogenous retinal detachment, IOP elevation, cataract and lens subluxation.^182^ Although rare (with a reported incidence of 0.09%‐0.87%),^183^ acute bacterial endophthalmitis is the most serious of these complications and requires immediate treatment with intravitreal antibiotics. Rates of vitreous haemorrhage,^118^ wound leak hypotony and retinal tears and detachment^123^ may be higher with DII, as the needle is larger (23‐gauge) and the force of injection greater than for standard 30‐gauge needle intravitreal injections. DII is contraindicated in aphakic and pseudophakic patients with posterior capsular rupture, as the implant may migrate into the anterior chamber.^184^ If this occurs, early removal is recommended to avoid chronic corneal oedema.^184^

### 5.2 Pharmacologic related complications

The two most important pharmacologic related complications of ocular steroids are raised IOP and development of cataract, which are more frequent with intravitreal injections compared to periocular injections.

#### 5.2.1 Raised IOP

Raised IOP with subsequent development of glaucomatous optic neuropathy is one of the most significant complications of locally administered corticosteroids. If other therapeutic options are available, ocular steroids are best avoided in patients with pre‐existing glaucoma. The pathogenesis is not well understood but may involve downregulation of trabecular meshwork matrix metalloproteinase activity, increased myocilin production and/or decreased trabecular meshwork phagocytic activity that increases aqueous outflow resisance.^185^ The susceptibility to pressure response may be due to genetic differences and variations in corticosteroid receptors whilst the degree of effect on IOP appears to be dose‐dependent.

##### Raised IOP with TA

In the SCORE Study Report 15, the proportion of patients being treated for BRVO or CRVO with a cumulative incidence of IOP elevation ≥10 mm Hg from baseline to 36 months was 2% (no IVTA), 9% (1 mg IVTA) and 45% (4 mg IVTA). Consideration of a lower dose (1 or 2 mg) of IVTA to treat RVO may be appropriate in patients at risk of an IOP‐related event, particularly as little difference has been reported in efficacy between the 1 and 4 mg doses.^22^, ^23^, ^185^ Other risk factors for an IOP‐related event include higher baseline IOP and younger age. A cumulative incidence of 32% of patients in the SCORE study reached an IOP ≥25mm Hg at 12 months. The incidence of IOP elevation in other reports is comparable.^186^, ^187^, ^188^ Although most cases of IOP elevation occur in the first 1 to 2 months of initiating therapy, in some cases it may take several months to develop (in SCORE Study Report 15 this ranged up to 598 days), so long‐term vigilance is required even if no IOP rise is seen after the first few injections.^185^

##### Raised IOP with DII

IOP elevation can also occur with DII, although possibly at lower rates than for IVTA. In the MEAD study for DMO, the incidence of IOP elevation ≥10 mm Hg from baseline to 36 months at any visit for those receiving DII 0.7 mg was 27.7%.^118^ The cumulative incidence of at least one visit with IOP ≥25 mm Hg or ≥35 mm Hg was 32% and 6.6%. The large majority of these patients with IOP elevation could be managed with medical therapy. Only one patient required incisional glaucoma surgery, and no patients required removal of the implant. Mean IOP returned to baseline by month 6 after each injection, and there did not appear to be a cumulative effect on IOP elevation with repeated injections. Similar findings were found in a 12‐month trial by the OZURDEX PLACID Study Group^189^ and a recent large retrospective analysis of 2736 eyes of 1441 patients treated with a total of 6015 DII in which 26.5% of eyes had an IOP rise >25 mm Hg but only 0.91% required glaucoma filtration surgery.^190^

##### Raised IOP with FA intravitreal implant

In the FAME trials, an IOP of ≥30 mm Hg developed in 16.3% of FA injectable implant treatment groups at month 23 and 18.4% by month 36. Elevated IOP that required incisional surgery by 36 months was 4.8% in the low‐dose group, 8.1% in the high‐dose group and 0.5% in the sham group.^191^

##### Raised IOP with periocular steroid

IOP elevation can also occur with periocular steroid. In a study by Sen et al^140^ of patients being treated with periocularly administered corticosteroid (predominantly TA 40mg) for uveitis, the cumulative incidence of at least 1 visit with IOP ≥24 mm Hg or ≥30 mm Hg at 12 months was 34% and 15%.

##### Raised IOP in uveitic patients

Corticosteroid‐induced raised IOP is much more common in the uveitic population than for other indications, and even higher in paediatric patients.^192^, ^193^, ^194^ In the POINT trial 20%, 30% and 41% recorded an IOP of ≥24 mm Hg by 24 weeks in the periocular, IVTA and OZURDEX groups, respectively, with 9%, 18% and 39%, respectively, developing an IOP rise of ≥10 mm Hg from baseline.^147^ Interestingly, only 4% to 6% overall developed an IOP of ≥30 mm Hg. Although both intravitreal treatments had a significantly higher rate of raised IOP when compared with periocular steroid, there was no significant difference seen between IVTA and OZURDEX in a direct comparison.

##### Raised IOP in children

Children are more likely than adults to have an IOP response to steroids.^195^ Compared to adult patients, the IOP rise can be more severe and resulting glaucoma may progress more rapidly and has even been reported within hours of starting treatment.^196^ The effect can result from topical, periocular, intravitreal, oral and intravitreal dosing regimes.

Dexamethasone is more likely to cause a steroid response than FML in children. A study by Kwok et al^195^ included 19 Chinese children undergoing bilateral strabismus surgery, with one eye randomized to receive topical dexamethasone 0.1% and the other to receive FML 0.1% six times per day for 4 weeks postoperatively. The mean increase in IOP in eyes receiving dexamethasone (15.48 ± 8.71 mm Hg) was almost double that of eyes receiving FML (5.83 ± 4.96 mm Hg; *P* = .001).^195^

FML also causes ocular hypertension, in a dose‐dependent manner in children. In a study by Fan et al,^197^ 31 children undergoing bilateral strabismus surgery had one eye randomized to receive topical FML six times per day and the other eye to receive topical FML three times per day, each for 4 weeks postoperatively. The IOP increased significantly from baseline in both groups, but the peak IOP was higher (19.0 ± 5.06 mm Hg vs 17.13 ± 3.32 mm Hg, *P* < .001) and the net increase was also greater (4.37 ± 4.79 vs 2.57 ± 3.32 mm Hg, *P* = .005) in the group with more frequent dosing. More frequent dosing was correlated with reaching peak IOP sooner (6 days vs 13 days; *P* = .033), but there was no difference in postoperative inflammation between the groups.^197^

#### 5.2.2 Cataract

Cataract is the other common complication of ocular and periocular delivered steroids. There are few studies that directly compare rates of cataract progression with different types of intravitreal steroids. However, the rates from various studies may suggest higher rates of cataract progression with IVTA and FA than DII. A head to head comparison between intravitreal FA and DII in uveitis patients showed a significantly higher incidence of cataract (and raised IOP) with FA.^198^

In the SCORE Study Report 5 (CRVO), the estimate through month‐12 of phakic patients developing new‐onset lens opacity or progression of existing opacity was 18%, 26% and 33%, respectively, in the observation, 1 mg IVTA and 4 mg IVTA groups. The percentage of patients requiring cataract surgery by 24 months in these respective groups were 0%, 0% and 33%.^22^ In the SCORE Study Report 6 (BRVO) this was 13%, 25% and 35%, respectively, in the laser, 1 mg IVTA and 4 mg IVTA groups.^23^ In the MEAD study for DMO, DII 0.7 mg was associated with cataract‐related adverse events in 67.9% of patients over the course of the 3‐year study, with 59.2% requiring cataract surgery during the study.^118^ A recent large retrospective analysis of over 6000 DII injections found a statistically significant increase in cataract progression in eyes receiving injections (*P* = .004) although with a small co‐relation co‐efficient (*r* = .057).^190^

In the FAME trials, the development of cataract and the proportion of patients requiring cataract surgery were significantly higher in both the low‐ and high‐dose FA treatment arms than in the sham‐control group. At 36 months, the percentage of patients developing cataracts were 81.7%, 88.7% and 50.7% in the low‐dose, high‐dose and control group, respectively.^199^

Uveitis patients may develop cataract from both intraocular inflammation and corticosteroid treatment. In the SITE cohort, 1192 eyes of 914 patients received periocular injections (sub‐Tenon's or orbital floor) for uveitis. Cataract development attributing to an incident reduction in visual acuity of worse than 6/12 occurred in 20.2%, whilst cataract surgery was performed within 12 months in 13.8% of initially phakic eyes.^140^

Periocular injection of corticosteroid used in the management of paediatric uveitis is associated with a high rate of cataract formation, [4 of 19 (21%) eyes]^200^ and IVTA used to manage uveitic macular oedema in children has been found to induce cataract in 6 of 11 eyes (55%).^145^

#### 5.2.3 Non‐infectious endophthalmitis and pseudoendophthalmitis

Although acute bacterial endophthalmitis is the most serious complication of intraocularly administered steroids, acute non‐infectious endophthalmitis following IVTA injection is more common, with most studies reporting an incidence of 0.5% to 2.0%.^201^, ^202^ Non‐infectious endophthalmitis refers to a transient, inflammatory reaction, typically with hypopyon that presents within a day or two post injection of IVTA.^201^ There is usually no or minimal inflammation of the sclera and conjunctiva, minimal anterior chamber fibrin and pain is rare, as might be expected since steroids are anti‐inflammatory.^201^ The condition is usually self‐limiting and resolves in 1 to 2 weeks but may cause persistent vitreous opacification in a minority of cases.

Some authors have attributed non‐infectious endophthalmitis following IVTA injection to the preservative vehicle 0.99% benzyl alcohol found in Kenacort.^202^ In one study, the incidence of severe sterile endophthalmitis fell from 13.0% to 4.3% after switching from preserved to preservative‐free TA.^203^ However another study found no difference in the incidence of non‐infectious endophthalmitis after removing benzyl alcohol^204^ and cases of non‐infectious endophthalmitis have been reported with benzyl‐alcohol free Triesence.^205^, ^206^ In fact, one study found a higher rate of non‐infectious endophthalmitis following administration of Triescence compared with Kenalog‐40 and preservative‐free TA.^207^ The authors attributed this result to the smaller particle size and higher particle load of Triesence. The pathogenesis for this phenomenon is likely to be multifactorial.

A non‐infectious, non‐inflammatory pseudoendopthalmitis can also occur when TA particles migrate and settle in the anterior chamber, resembling a hypopyon.^201^ Whenever there is uncertainty regarding whether an endophthalmitis is infectious or non‐infectious, it should be managed with the standard treatment for infectious endophthalmitis.

#### 5.2.4 Activation of ocular or periocular infection

Corticosteroids may suppress the host response and thus increase the hazard of secondary ocular infections. This can prolong the course and/or exacerbate the severity of viral (eg, herpes simplex epithelial keratitis), bacterial and fungal infections of the eye. In acute purulent infections of the eye, steroids may mask infection or exacerbate existing infection. Since corticosteroids are known to reduce resistance to infections, simultaneous bilateral intraocular injections of steroids should, if possible, be avoided to limit the potential for bilateral postoperative infection.^208^

Infectious scleritis is a rare complication of sub‐Tenon TA with a reported incidence of 0.04%.^209^ A recent review found nine reported cases in which four were caused by fungal organisms.^210^

Use of intraocular or periocular administered corticosteroids may reactivate HSK and concurrent antiviral prophylaxis for those with a history of HSK is advised.^211^, ^212^

Viral retinitis has been reported following local administration of corticosteroids. A recent review found 30 cases with causative viruses identified as cytomegalovirus in 76.7%, HSV in 16.7%, with one case of varicella zoster virus and another unspecified. Steroids were administered by IVTA (33.3%), intravitreal FA acetonide implant Retisert (33.3%), posterior sub‐Tenon TA (6.6%) and anterior sub‐Tenon TA (3.3%).^208^

Unmasking or activation of dormant ocular syphilitic infection following IVTA have been reported in five cases. All cases manifested with small round yellow retinal lesions, retinovasculitis and optic disc involvement.^213^, ^214^, ^215^ Three of these cases presented with acute syphilitic posterior placoid chorioretinitis and all had devastating visual outcomes.^213^, ^214^

In contrast, a small series of seven patients demonstrated safety of DII in treating CMO in patients with infectious uveitis where other treatments for CMO had failed. All patients were concurrently treated on the appropriate antimicrobial agent and had resolution of CMO without reactivation of the infectious ocular disease.^216^

#### 5.2.5 Steroid induced central serous chorioretinopathy

It is well established that any exogenous use of corticosteroids may precipitate or exacerbate central serous chorioretinopathy (CSC).^217^ Despite topical, periocular and intravitreal steroids being common ophthalmic treatments, CSR is rarely reported in association with administration of ophthalmic steroids.^218^

A recent literature review found only one case of IVTA causing new‐onset CSC,^219^ and another case of worsening pre‐existing CSC.^220^ Interestingly, a reported case of intravitreal Ozurdex implantation for refractory Irvine‐Gass syndrome was found to precipitate CSC in the fellow eye.^221^ One case presenting with bilateral multifocal serous retinal detachments initially diagnosed as Vogt‐Koyanagi‐Harada syndrome received high‐dose oral steroids and IVTA. The treatment ultimately worsened the condition later diagnosed as CSC.^222^

In contrast, IVTA experimentally used to treat chronic CSC and CMO secondary to CSC, did not improve or worsen the CSC.^223^, ^224^ Surprisingly, a recent literature review found only one report of CSC associated with periocular steroid use,^225^ and no cases of CSC related to topical steroid eye drops.^218^

**Table 3:** Outline of studies on local delivery of corticosteroids in clinical ophthalmology

Conditions	Authors, (Year)	Design	No. of eyes/(patients)	Treatment steroid	Study aims and outcome measures	Conclusions
*Adnexal*						
TED	Bordaberry et al, (2009)^31^	RCT	21	Peribulbar TA	To assess the efficacy of peribulbar TA to treat inflammatory signs of moderate to severe Graves' orbitopathy and associated optic neuropathy Clinical activity score Extraocular muscle size	Peribulbar TA reduced inflammatory signs of moderate Graves' orbitopathy as measured by the clinical activity score
	Ebner et al, (2004)^32^	Multi‐centre RCT	41	Peribulbar TA	To assess the efficacy of peribulbar TA vs control to treat TED Extraocular muscle size Binocular diplopia	Peribulbar TA is effective in reducing diplopia and extraocular muscle size in TED
	Alkawas et al, (2010)^226^	RCT	12	Peribulbar TA Oral Prednisolone	To assess the efficacy of peribulbar TA vs oral prednisolone to treat TED Modified clinical activity score Signs/Exophthalmometry Complications	No statistical difference found in study sample between peribulbar TA and oral prednisolone in treating TED
	Lee et al, (2013)^35^	Single‐blinded RCT	(106)	Sub‐conjunctival TA	To assess the efficacy of sub‐conjunctival TA in treatment of TED related lid retraction Lid retraction Lid swelling Exophthalmos Lagophthalmos Clinical activity score Total eye score	Sub‐conjunctival TA was effective in treating TED related lid retraction and persisted through to 24 weeks of follow‐up
Nasolacrimal disease	McNeill et al, (2005)^44^	RCT	11	Nasal beclomethasone	To assess the efficacy of nasal corticosteroids in treating functional epiphoria in patients with rhinitis Epiphoria symptom score	Epiphoria secondary to rhinitis can be treated successfully with intranasal beclomethasone
Chalazia	Goawalla and Lee, (2007)^46^	RCT	136	Intra‐lesional TA Incision and curettage Hot compresses	To compare intra‐lesional TA, incision and curettage and hot compresses in the treatment of chalazia Resolution rate Pain/inconvenience score Patient satisfaction score	Resolution rates between intra‐lesional TA and incision and curettage were similar and both were significantly greater than conservative group. There was less pain and patient inconvenience with intra‐lesional TA compared to incision and curettage
	Ben Simon et al, (2011)^47^	RCT	94	Intra‐lesional TA Incision and curettage	To compare intra‐lesional TA against incision and curettage for the treatment of chalazia Resolution rate Additional treatments	Intra‐lesional TA was as effective as incision and curettage in primary chalazia
*Anterior segment*						
Bacterial keratitis	Srinivasan et al, (2012)^60^	Multi‐centre placebo‐controlled double‐blinded RCT	500 (3 mo) 399 (12 mo)	Topical prednisolone Placebo	To compare the benefit in clinical outcomes of adjunctive topical corticosteroids in the treatment of bacterial corneal ulcers BCVA Infiltrate/scar size Re‐epithelialisation Corneal perforation	No significant differences in clinical outcomes with topical prednisolone sodium phosphate 1% compared to placebo in non‐Nocardia species. Ulcers caused by Nocardia may fare worse with topical steroids
HSK	Wilhelmus et al, (1994)^62^	Multi‐centre placebo‐controlled double‐blinded RCT	106	Topical prednisolone Placebo	To compare the benefit in clinical outcomes of adjunctive topical corticosteroids in the treatment of HSV keratitis Clinical resolution	Topical prednisolone phosphate was significantly better than placebo in reducing persistence or progression of stromal inflammation (by 68%)
Allergic eye disease	Singh et al, (2001)^69^	Double‐blinded RCT	90 (45)	Supratarsal DM Supratarsal TA Supratarsal HC	To compare three types of supratarsal steroid injections for the treatment of refractory VKC Symptoms and signs (cobblestone papillae, lid oedema, conjunctival discharge, chemosis, Horner‐Tranta dots and shield ulcers) Disease recurrence	All three drugs were equally effective with no statistically significant difference in the time of resolution. Recurrence was seen within six in all cases irrespective of the steroid used
	Saini et al, (1999)^70^	Double‐blinded RCT	38 (19)	Supratarsal TA Supratarsal DM	To compare supratarsal TM vs supratarsal DM for the treatment of refractory VKC Symptoms and signs (cobblestone papillae, lid oedema, shield ulcer, SPK) Disease recurrence	Both were equally effective in controlling symptoms and signs however supratarsal TM had a lower recurrence rate
KCS	Pflugfelder et al, (2004)^81^	Multi‐centre double‐blinded RCT	128 (64)	Topical loteprednol etabonate 0.5% Placebo	To assess the efficacy of loteprednol etabonate 0.5% vs placebo for KCS Symptom severity score Corneal fluorescein staining Conjunctival injection	Topical loteprednol etabonate may be beneficial in KSC with moderate clinical inflammation
	Sheppard et al, (2014)^84^	Multi‐centre double‐blinded RCT	(119)	Topical loteprednol etabonate 0.5% + topical cyclosporine 0.05% Topical cyclosporine 0.05% + artificial tears	To assess the efficacy of loteprednol etabonate 0.5% with topical cyclosporin 0.05% in dry eye disease Ocular surface disease index (OSDI) Likert scale Lissamine green staining, fluorescein staining, Schirmer test	Loteprednol showed greater efficacy in dry eye signs and symptoms than topical cyclosporin or artificial tears alone. It also provided rapid relief of dry eye disease
	Lin and Gong, (2015)^86^	Multi‐centre double‐blinded RCT	(41)	Topical FML 0.1% Cyclosporine A 0.5%	To compare topical FML vs cyclosporin A for the treatment of dry eye disease in Sjogren syndrome Fluorescein staining OSDI Conjunctival goblet cell density Severity of conjunctival congestion Tear break up time (TBUT)/Schirmer test	Both medications gave similar improvement from baseline, however topical FML provided faster improvement in symptoms of ocular dryness
	Pinto‐Fraga et al, (2016)^87^	Double‐blinded RCT	(42)	Topical FML 0.1% Artificial tears	To assess the efficacy of topical FML in dry eye disease when exposed to adverse environments Corneal and conjunctival staining Conjunctival hyperaemia TBUT Tear osmolarity Symptom assessment in dry eye (SANDE)	Topical FML was effective in alleviating dry eye disease but also especially in preventing exacerbation caused by exposure to a desiccating stress
GVHD	Yin et al, (2018)^89^	Double‐blinded RCT	42	Topical loteprednol 0.5% Artificial tears	To assess the efficacy of topical loteprednol in dry eye disease associated with GVHD OSDI Corneal fluorescein staining Conjunctival lissamine green staining TBUT Schirmer test	Topical loteprednol had a less favourable response in treating dry eye disease in GVHD compared to those without GVHD
Chemical Injury	Brodovsky et al, (2000)^93^	Retrospective series	177 (121)	Intensive Topical FML + treatment protocol Conservative management	To compare treatment outcomes of a standard protocol of intensive treatment vs conservative management in alkali‐burned corneas Time to corneal re‐epithelialisation Final BCVA Time to visual recovery Length of hospital stay Complications	Patients with intensive treatment had a trend for rapid healing and better final visual outcomes in grade 3 chemical burns but no difference in grade 4 burns
Anterior scleritis	Sohn et al, (2011)^13^	Retrospective multi‐centre cohort	68 (53)	Sub‐conjunctival TA	To assess the efficacy of sub‐conjunctival TA for non‐necrotising anterior scleritis Resolution of symptoms and signs Recurrence Adverse effects	After one injection sub‐conjunctival TA gave improvement of symptoms and signs in 97% and eyes remained recurrence‐free in 67.6% at 24 mo. Sub‐conjunctival TA is a useful adjuvant therapy that may reduce the burden of systemic medication
*Glaucoma surgery*						
Glaucoma filtration surgery	Araujo et al, (1995)^100^	RCT	46 (35)	No corticosteroids Topical 1% prednisolone acetate Topical 1% prednisolone acetate and oral prednisone	To compare no adjunctive steroids vs topical prednisolone vs topical prednisolone and oral steroids in glaucoma filtration surgery after 10 y Final IOP in follow‐up periods Number of glaucoma medications used Additional glaucoma filtration surgery Visual acuity Stabilization of glaucoma (disc photos, visual fields)	Patients treated with steroids (groups 2 and 3) had significantly improved outcomes compared with patients without steroids (group 1). Group 1 had more additional procedures, higher IOPs, more additional glaucoma drops and lower rate of stabilized glaucoma
	Yuki et al, (2009)^102^	RCT	53	Sub‐Tenon TA Control (no TA)	To assess the efficacy of intraoperative sub‐Tenon TA on the success rate of trabeculectomy in secondary glaucoma IOP reduction Success rate Indiana Bleb Appearance Grading Scale	Intraoperative sub‐Tenon TA neither increased intermediate‐term success nor decreased postoperative complications
	Breusegem et al, (2010)^227^	RCT	54	Topical FML Topical ketorolac Placebo	To compare preoperative treatment of topical ketorolac or FML vs placebo on trabeculectomy outcomes Postoperative surgical or medical interventions (needling, suture lysis, needling revision, IOP‐lowering medication)	Use of topical ketorolac or fluorometholone 1 mo prior to trabeculectomy was associated with less likelihood of postoperative needling and less need for IOP‐lowering medication
	Yazdani et al, (2017)^110^	Triple‐blinded RCT	90	Sub‐Tenon TA Placebo	To compare intraoperative sub‐Tenon TA vs without in Ahmed glaucoma valve implantation IOP BCVA Occurrence of hypertensive phase Peak IOP Number of glaucoma medications Postoperative complications	Sub‐Tenon IOP resulted in a lower mean IOP at the first mo and was 1.5 mmHg lower throughout the study period. Peak postoperative IOP was also lower. The rates of success, occurrence of hypertensive phase and complications were similar between the two groups
*Posterior segment*						
DMO	Gillies et al, (2006)^115^	Double‐blinded RCT	69 (43)	IVTA Placebo	To assess the efficacy of outcomes of IVTA in the treatment of refractory DMO Improvement of BCVA Central macular thickness Adverse events	IVTA had significantly greater proportion of patients (56%) achieving ≥15 letters of improvement in BCVA than placebo (26%). IVTA was also found to reduce central macular thickening however adverse events included cataract and glaucoma
	Bressler et al, (2010)^117^ DRCR Protocol I	Multi‐centre double‐blinded RCT	828	Ranibizumab + Prompt laser Ranibizumab + deferred laser Prompt laser + sham IVTA + prompt laser (Latter two groups allowed very deferred ranibizumab)	To compare intravitreal ranibizumab plus prompt or deferred laser vs prompt laser or IVTA plus prompt laser in DMO Improvement of BCVA Central subfield thickness Number of injections (5 y)	Eyes receiving initial ranibizumab for centre‐involving DMO had better long‐term vision and reduced central subfield thickness
	Boyer et al, (2014)^118^ MEAD study	Two identical, parallel multi‐centre double‐blinded RCT	(1048)	DII (0.7 mg) DII (0.35 mg) Sham	To assess safety and efficacy of DII in the treatment of DMO Improvement of BCVA Central retinal thickness Adverse events	DII had significantly greater proportion of patients achieving ≥15‐letters of improvement in BCVA (22.2% for 0.7 mg, 18.4% for 0.35 mg and 12.0% for sham)
	Fraser‐Bell et al, (2016)^120^ BEVORDEX study	Multi‐centre single‐blinded RCT	88 (61)	DII Bevacizumab	To compare DII vs intravitreal bevacizumab for the treatment of DMO Improvement of BCVA CMT Injection frequency Adverse events	DII achieved similar rates of BCVA improvement with bevacizumab and superior anatomic outcomes with fewer injections at 12 mo. At 24‐mo, there was no significant difference of improvement in BCVA but less burden of injections
	Callanan et al, (2013)^189^	Double‐blinded multi‐centre RCT	253	DII + laser Laser	To compare DII combined with laser photocoagulation compared with laser alone for treatment of diffuse DMO BCVA Vessel leakage Adverse events	DII combined with laser resulted in significantly greater mean improvement in BCVA at all time points through month 9. Combination treatment also reduced areas of diffuse vascular leakage on angiography. At 12 mo, there was no significant difference between the two groups
	Campochiaro et al, (2011)^121^ FAME A and B	Two identical parallel, multi‐centre double‐blinded RCT	392	IVFA implant (0.2 μg/d) IVFA implant (0.5 μg/d) Sham	To compare efficacy and safety of IVFA implants for treatment of DMO Improvement of BCVA Foveal thickness Adverse events	Low‐dose and high‐dose IVFA implant groups had greater percentage of patients with ≥15 letters of improvement in BCVA at 24 mo (28.7% and 28.6%) compared with sham (16.2%). There was also more improvement in foveal thickness compared to sham. A significant percentage (7.6%) of the high‐dose group required incisional glaucoma surgery
CMO in RVO	Ip et al, (2009)^22^ SCORE‐CRVO	Multi‐centre RCT	271	IVTA 1 mg IVTA 4 mg Standard of care (observation)	To assess the efficacy and safety of IVTA for treatment of macular oedema secondary to central retinal vein occlusion Improvement of BCVA Centre point thickness Vessel leakage, capillary non‐perfusion Adverse events	IVTA had significantly greater proportion of patients with ≥15 letter improvement in BCVA (27% for 1 mg, 26% for 4 mg and 7% for sham). Superior safety profile of 1 mg dose compared with 4 mg dose IVTA with respect to glaucoma and cataract
	Scott et al, (2009)^23^ SCORE‐BRVO	Multi‐centre RCT	411	IVTA 1 mg IVTA 4 mg Standard of care (grid laser)	To assess the efficacy and safety of IVTA for treatment of macular oedema secondary to branch retinal vein occlusion Improvement of BCVA Centre point thickness Vessel leakage, capillary non‐perfusion Adverse events	Treatment with IVTA with 1 mg or 4 mg or standard of care did not demonstrate a significant difference in visual acuity outcomes in macular oedema secondary to branch retinal vein occlusion
	Haller et al, (2010)^123^ GENEVA	Two identical, parallel multi‐centre double‐blinded RCT	1267	DII Sham	To assess the efficacy for DII for treatment of macular oedema secondary to CRVO or BRVO Improvement of BCVA Central retinal thickness Adverse events	DII had significantly greater proportion of patients with ≥15 letter improvement in BCVA, mean BCVA and less proportion of patients losing ≥15 letters in BCVA
Posterior non‐infectious uveitis	Sen et al, (2014)^140^ SITE	Retrospective review of multi‐centre cohort	1192 (914)	Periocular corticosteroid (including sub‐Tenon and orbital floor)	To assess the efficacy and safety of periocular corticosteroid injections in uveitis Improvement of BCVA Improvement of macular oedema affecting BCVA Intraocular inflammation Systemic medications Adverse events	Over 50% of eyes demonstrated improved VA at some point within 6 mo of receiving periocular steroid. Periocular corticosteroids were also effective in treating acute inflammation or macular oedema
	Kempen et al, (2015)^141^ MUST study	Multi‐centre RCT	479 (255)	IVFA implant (Retisert) Systemic therapy	To compare IV FA implant with systemic immunosuppression in the treatment of posterior non‐infectious uveitis BCVA Visual field mean deviation Activity of uveitis Presence of macular oedema	No significant difference in BCVA at 2 and 5 y. Systemic immunosuppression had better BCVA outcome at 7 y
	Jaffe et al, (2019)^30^	Multi‐centre, double‐blinded sham‐controlled RCT	129	IVFA implant (Yutiq) Sham	To assess efficacy and safety of IVFA implant on recurrence rates in chronic posterior non‐infectious uveitis Improvement of BCVA Recurrence of uveitis Macular oedema Adverse events	IVFA provided a greater proportion of patients with ≥15 letter improvement as well as effective management of intraocular inflammation and lower recurrence rates during the first 12 mo
	Lowder et al, (2011)^146^ HURON study	Parallel‐group, multi‐centre, blinded RCT	229	DII (0.7 mg) DII (0.35 mg) Sham	Efficacy of DII on treating inflammation and CMO in non‐infectious posterior uveitis or panuveitis Vitreous haze BCVA Central macular thickness Adverse events	Both doses of DII showed a significant reduction of posterior inflammation and CMO compared to sham which persisted through week 25. The proportion of patients with ≥15 letter improvement of BCVA was also significantly higher in the DII groups compared to sham
	Thorne et al, (2019)^228^ POINT trial	Multi‐centre, parallel‐treatment comparative RCT	235	Periocular TA IVTA DII	To compare the efficacy of periocular TA, IVTA and OZURDEX for treatment of uveitic macular oedema Central subfield thickness Resolution macular oedema BCVA IOP events	Improvements of CMT were seen in all three groups, periocular TA (23%), IVTA (39%), OZURDEX (46%). Greater improvements in BCVA were also seen with IVTA and OZURDEX. No significant differences between IVTA and OZURDEX in central subfield thickness or BCVA
	Das et al, (1999)^151^	RCT	63	IVDM + intravitreal antibiotics Intravitreal antibiotics alone	To compare adjunctive IVDM vs intravitreal antibiotics‐only during vitrectomy for suspected postoperative or post‐traumatic bacterial endophthalmitis Inflammation scoring BCVA	A reduction of inflammation was observed in the IV DM group at 1 week and 1 mo (although topical corticosteroids were not given in the intravitreal antibiotic‐only group). Final visual outcomes at 3 mo were not significantly different
	Gan et al, (2005)^152^	RCT	29	IVDM + intravitreal antibiotics Intravitreal antibiotics alone	To compare adjunctive IVDM vs intravitreal antibiotics alone in postoperative endophthalmitis BCVA	No statistically significant difference on visual acuity at 3 and 12 mo between the two groups. Trial terminated prematurely due to the study drug (dexamethasone sodium diphosphate was no longer available)
	Albrecht et al, (2011)^153^	Double‐masked RCT	62	IVDM + intravitreal antibiotics Intravitreal antibiotics alone	To compare adjunctive IV DM vs intravitreal antibiotics alone in presumed bacterial endophthalmitis BCVA	No statistically significant difference in visual outcomes in short‐term (2 weeks) or intermediate‐term (2–4 mo post‐treatment) between the two groups
	Manning et al, (2018)^154^	Multi‐centre RCT	167	IVDM + intravitreal antibiotics Intravitreal antibiotics alone	To compare adjunctive IVDM vs intravitreal antibiotics alone in patients with suspected bacterial endophthalmitis post‐cataract surgery BCVA	No statistically significant difference in final visual outcomes between IVDM and placebo group
Postoperative CMO	Konstantopoulos et al, (2008)^158^	Retrospective case series	21 (20)	IVTA (4 mg)	To assess efficacy and safety of IVTA in postoperative CMO Improvement of BCVA Adverse events	All patients had significantly improved BCVA from baseline which was maintained at 6 mo
	Thach et al, (1997)^229^	Retrospective review	49 (48)	Retrobulbar TA (40 mg) Posterior sub‐Tenon TA (40 mg)	To compare the retrobulbar TA vs posterior sub‐Tenon's TA for pseudophakic CMO refractory to topical medications BCVA Resolution of CMO IOP	There was significant improvement in BCVA compared to baseline for both groups but no statistically significant difference was found between the two groups

Abbreviations: CMO, cystoid macular oedema; DM, dexamethasone; DMO, diabetic macular oedema; FML, fluoromethalone; HC, hydrocortisone; HSK, herpes simples keratitis; IV, intravitreal; JXH, Juvenile xanthogranuloma; KCS, keratoconjunctivits sicca; LCH, Langerhan's cell histiocytosis; MP, methylprednisolone; PA, prednisolone acetate; RCT, Randomized Controlled Clinical Trial; RVO, retinal vein occlusion; TA, triamcinolone; TED, thyroid eye disease; VKC, vernal keratoconjunctivitis.

## 6 CONCLUSION

For over 70 years, ocular steroids have proven to be a major weapon in the treatment of ocular inflammation. An increasing variety of preparations and delivery sites have maximized their efficacy whilst attempting to minimize their major side effects‐ raised IOP and cataract. Multiple RCTs have improved our understanding of the utility and limitations of this versatile medication.

## FINANCIAL DISCLOSURE

Adrian Fung, Honoraria and travel support: Allergan, Bayer, Novartis. Lyndell Lim, Research grant: Abbvie, Bayer. Advisory Board work and consultant fees: Bayer, Abbvie, Allergan. Chameen Samarawickrama, Research funding: Westmead Charitable Trust Early Career Research Fellowship. Jennifer Arnold, Advisory Board work and honoraria: Allergan, Novartis, Bayer, Alcon. Mark Gillies, Research funding, honoraria and travel support: Novartis, Bayer, Allergan. Andrew Symons, Research grant: Topaz Study.
